# Our Children’s Eyes and Books

**Published:** 1884-07

**Authors:** F. C. Hotz

**Affiliations:** 181 Clark Street; Chicago


ARTICLE II.

### OUR CHILDREN'S EYES AND BOOKS. *By* F. C. HOTZ, M. D. *Chicago.*

A child is seldom born near-sighted, and an adult past the twentieth year scarcely ever gets near-sighted, though his occupation (as watchmaker, for instance) may demand the constant use of his eyesight at a very short range. But although the most children enter school with good eyesight, the experience all over the world has shown that a great many children leave the school near-sighted. Within the past ten or twelve years numerous examinations of the eyes of school children (between six and sixteen years) were made in every country of the civilized world; and whether the reports come from Germany or France, from England or Russia, from Switzerland or our own country, all the statistics prove that there is more or less near-sightedness in every nation, and that the percentage of near-sighted pupils increases in all countries (in some countries at a frightful rate), from the lower to the higher grades in each school, and from the common schools to the high schools and colleges. On the other hand, it is a fact that near-sighted eyes are not found among the uncivilized races.

These observations and facts have lead to the conclusion that there exists an intimate causal relation between the occupations of our children during the period of school life and the development of near-sightedness, and that reading and writing are the occupations which, under certain conditions, act so injuriously upon the eyes of children. The sclerotic of their eyes is thin and extensible; its form, therefore, may possibly be influenced by tension and pressure; and as the size of the eyeball is principally determined by the shape of its sclerotic, the same forces which can change the shape of the latter will necessarily change the length and breadth of the eyeball.

During reading and writing we must adjust the focus and the visual axis of our eyes to the distance at which the book is placed. Both these adjustments (accommodation and convergence) are executed by the contraction of muscular fibres (the ciliary and recti muscles), and it is supposed that by the long-continued and oft-repeated tension and pressure upon the eyeball, associated with these muscular contractions during accommodation and convergence, the extensible sclerotic of a child's eye can be stretched and the eyeball be elongated, and so become near-sighted.

Here the question might be asked, If this theory be correct, why do not all eyes become near-sighted, as all school children have to read and write? This question can be answered with the same explanation which we give in accounting for the fact that some children's health suffers seriously by the same amount of school work which other children can do with the greatest ease and comfort. We speak of robust and delicate constitutions to indicate the different degrees of endurance for physical or mental work which different individuals possess; and we may with the same propriety speak of robust and delicate eyes to explain the different effect the same amount of school work produces upon them. A child with robust eyes will at the end of his school period still enjoy perfect eyesight, while the delicate eyes will break down under the same amount of work and become near-sighted.

The degree of endurance of the eye, the greater or less tonicity of its tunics, is largely dependent upon the constitutional character of the whole organism. The robust children, as a rule, are blessed with good and strong eyes, while the weak and delicate constitutions furnish the feeble and easily extensible sclerotics of the myopic eyes. And when we are informed by the statistician that the percentage of near-sighted school children is much larger in cities than in country districts, this is exactly what we should expect to find according to the constitutional differences between city and country children.

If we recognize the important role the constitutional infirmity of a child plays as a predisposing cause of myopia, if we believe the eyes of weakly constituted children are so delicately constructed that their natural growth can be easily disturbed and their shape be altered by the ordinary amount of school work, we understand at once why near-sighted children are found in every civilized country. Our children must get a proper school education; they must read and write; they must apply their eyes upon books; and since in every community there is always a number of delicate children whose eyes cannot endure the efforts of accommodation and convergence, they are doomed to become near-sighted.

Myopia, therefore, is in a certain sense the logical result, the inevitable evil, of our civilization, and will exist as long as this lasts. But while we cannot hope for the complete extermination of this evil, because the predisposing causes are beyond our control, we may expect to mitigate it and to reduce the number of its victims, because the immediate causes of myopia (accommodation and convergence) can be regulated and controlled; for many children who otherwise would grow up to manhood with perfect eyesight become near-sighted because they are allowed to read under disadvantages which overstrain the muscles of accommodation and convergence. All these eyes could be saved by a little care and attention. We should see that our children do not read too long; we should see that they do not read by insufficient light, and we should see that they do not approximate the book too much.

Whatever is said about reading applies to the act of writing, too; but of the two occupations I regard reading as having the greater influence upon the development of myopia. Unquestionably, a child reads a great deal more than it writes; and when it reads an interesting book its eyes are steadily fixed upon the page and never allowed to rest except when the leaf is turned; while in writing, the frequent interruptions for filling the pen afford to the eyes as many occasions for relaxing the muscles of accommodation and convergence. If, therefore, the continuous and excessive efforts of these muscles produce myopia, they are more likely to produce the injury during the act of reading than during writing.

Children have a natural tendency to hold small objects unnecessarily near their eyes. This tendency is the principal source of danger for eyes disposed to become near-sighted; and parents and teachers cannot be too careful lest this tendency to approximate the book grows into a bad and dangerous habit. Since our children cannot get a proper education without reading and writing; since they must read, we should at least see that they do it under conditions which put the least possible strain upon their eyes. It is, therefore, of the utmost importance to know that eighteen to twenty inches are considered the shortest distance at which children may safely be allowed to read. For this distance the convergence of the visual axis is so slight and the amount of accommodation so small that the muscles of the eyes are not taxed severely. But with every inch by which this distance is shortened the work imposed upon the ocular muscles rapidly increases, and soon causes a degree of tension and pressure dangerous to the soft tunics of a child's eye.

But if we do not want our children to read at a distance less than eighteen inches we must not let them have books with print which it is next to impossible for them to read at the required distance. *Javal, Weber, Cohn,* and others who have written on this question of book print, declared that books should not be printed in letters smaller than 1.50 millimeter, and some experts put the limitation even at 1.75 millim. for children's books. Easy and comfortable reading furthermore requires that the lines should be well "leaded;" in other words, there should be an interval of space between the lines of no less than 2.50 to 3 millim.; and it is hardly necessary to mention that the paper must be smooth, firm, and thick to show a clean and clear print. A beautiful specimen of good book print, which can be highly recommended to all publishers, is Solis Cohen's work on "Diseases of the Nose and Throat" (size of letters 1.75 m.). If the reader compare that print with print of this Journal (size of letters 1.50 m.), or with that of the *Chicago Tribune* (local columns 1.00 m.), he will realize the difference between proper and improper print; and by the different effects they produce in his own eyes he may judge what the effect must be upon the tender eyes of children. And he will at once be interested to know what sort of print our children read in school and at home.

To answer this question, I have examined the most books used in the Grammar and High Schools of Chicago; also a number of books at the Public Library which were pointed out as much read by boys and girls; and finally, the magazines for young folks. This examination has brought out some very interesting facts.

Of ten books of the Grammar Schools all but one reached the standard. This one exception is the "School Dictionary;" the words are printed in *agate* (1.00 mill.) and the definitions in pearl (0·75)! and one millim. is allowed between the lines. Just think of the contrast! Now the child's eyes are resting on the large, clear, and easy print of its Reader, and the next minute they must accommodate themselves to the blurred microscopic letters of this Dictionary. These sudden changes of the accommodation, repeatedly made in short intervals, are a fruitful source of spasm of accommodation among children. If any fiend attempted to ruin the eyes of our children he could not hit upon a better device than this School Dictionary. It cannot be condemned in too severe language, and no school board with a heart for the welfare of the children should tolerate its use in the schools. If the teachers cannot give the proper definitions of such words as children eight or nine years old use and read, if young children must use a dictionary, let them at least have one with decent type.*

The fourteen High School books I examined make a very good record; all have good paper, the text is printed in letters of 1.75 mill. in ten and 1.50 mill. in four books; the interlinear space 3 mill. in six, 2.50 in eight; only in the notes the type is reduced to 1.25 mill.

In the twenty-three books of Fiction at the Public Library the paper was not of uniform quality; in ten it was good; in eight fair, and in five positively poor. *None* of the books had letters of 1.75 mill.; in seven only they measured 1.50, while in sixteen (that is in the majority) the size was 1.25 mill. The width of the interlinear space varied all the way from 2 to 3.75 mill., but in the majority it ranged between 2.25 and 2.75 mill.

Highly interesting is the examination of the magazines and papers for the young people. Ten periodicals were examined;

---

* In this connection let me call the attention also to the small Bibles commonly used by children in the Sunday schools. The letters of the text measure 0·75 millim., and the italics of the superscriptions of the chapters are still smaller. The space between the lines is one millim. wide, and the paper is of so much inferior quality that the print shows through the paper. It is the worst kind of book print I ever saw, and what I have said concerning the School Dictionary applies even with greater force to this kind of Bible. Further comment is unnecessary.

the paper was found good in four, fair in two and poor in four. The letters measured 1.75 in but one; 1.50 in three; 1.25 in five, and 1.00 in one. The interlinear space was 3.25 in only one paper, in all the rest it varied between 1.00 and 2.25 mill. There was but one paper among the eleven which attained the standard of good paper, proper size of type and proper leading. And the magazine whose letters dwindled down to 1 mill. (agate) and whose lines are separated only by 1 mill. space is one of the most popular, if not *the* most popular paper read by our girls and boys.

In reading this report we cannot fail to notice the great difference in quality of print between the school books on the one side, and the books and periodicals the children read at home. The superiority of the school books is worthy of special notice. For it has been a common practice to charge the schools and school books with being the principal causes for the development of myopia. Much stress has been laid upon the injurious effect of insufficient or faulty illumination of school-rooms; much has been written about unsuitable school desks and seats, and grand results were confidently expected from a reform of the school rooms. But the results were sadly disappointing. The statistics showed that in the best arranged schools the number of myopic children was not smaller, nay, sometimes was found even larger than in similar schools with inferior appointments. Like the man who sees the mote in his neighbor's eye, but not the beam in his own, we were quick in exposing the faulty hygiene of the school room, but have paid little or no attention to the great and glaring offenses which were daily committed against the same hygienic principles in our homes. When our children get near-sighted, the mischief is not done principally in the schools, but at home! Our school rooms are well lighted; our school desks and seats are acknowledged, the world over, as models of simplicity and perfection; and the desks are placed in proper relation to the windows. And as my examinations of the school books show, they, too, stand the test admirably well; all (with one exception, which I have noted,) fulfill those requirements which we regard of paramount importance in a book for the use of children.

But now let us look to the homes! How many houses afford the children as well lighted rooms as their school room? The rooms of the rich are dimmed by curtains and shades, and the rooms of the poor are dark for want of large windows, or because they are situated in basements or narrow alleys. How many children have a suitable desk and chair at home? How many parents take care that their children do not read or write with the light falling on the book from the wrong side, or in the evening seated too far away from the gas light? And how many books the children read at home can compare typographically with their school books? As our statistics show, only seven out of twenty-three books, and only one out of ten periodicals! But if the prolonged application of the eyes of children is the chief cause of making eyes so disposed near-sighted, upon which books do our children fix their eyes more steadily, more attentively, and more uninterruptedly? On the school books which they are obliged to read, or on the story books which they read for pleasure? Which, consequently, are likely to do the more harm to their eyes, the bold letters of the Fourth Reader, or the blurred nonpareil of the "Arabian Nights;" the clear brevier type of "Shaw's History of English Literature," or the fascinating stories of the "Waverly Magazine" with its agate type?

When we look at a child deeply absorbed in a story book; when we see how the little face gradually approaches and almost touches the page; and when we then think of the enormous muscular strain these young eyes have to endure, in order to read the miserable print, then we shall know better at whose door we have to lay the responsibility for the impaired eyesight of so many children. Let parents understand that the hygienic principles they are so anxious to see observed in the schools must just as strictly be enforced in their own house. Let them adopt the typographical standard of the school books for all the other books their children may read, and there will be no place for penny papers, dime libraries and all the rest of the so-called cheap literature. It is the dearest sort of reading matter people can buy for their children; it is a penny-wise-and-pound-foolish domestic policy; they are saving the pennies but wasting that which once lost no money in the world can restore—perfect eyesight.

181 Clark Street.

